# Biological Implications of a Stroke Therapy Based in Neuroglobin Hyaluronate Nanoparticles. Neuroprotective Role and Molecular Bases

**DOI:** 10.3390/ijms23010247

**Published:** 2021-12-27

**Authors:** María Ángeles Peinado, David Ovelleiro, María Luisa del Moral, Raquel Hernández, Esther Martínez-Lara, Eva Siles, José Rafael Pedrajas, María Luisa García-Martín, Carlos Caro, Sebastián Peralta, María Encarnación Morales, María Adolfina Ruiz, Santos Blanco

**Affiliations:** 1 Department of Experimental Biology, Campus de Las Lagunillas s/n, University of Jaén, Building B3, 23071 Jaen, Spain; dovelleiro@gmail.com (D.O.); mlmoral@ujaen.es (M.L.d.M.); rhernand@ujaen.es (R.H.); elara@ujaen.es (E.M.-L.); esiles@ujaen.es (E.S.); pedrajas@ujaen.es (J.R.P.); 2 BIONAND-Centro Andaluz de Nanomedicina y Biotecnología, Junta de Andalucía, Universidad de Málaga, Parque Tecnológico de Andalucía, 29590 Malaga, Spain; mlgarcia@bionand.es (M.L.G.-M.); cacarsal@gmail.com (C.C.); 3 Department of Pharmacy and Pharmaceutical Technology, Campus de Cartuja s/n, School of Pharmacy, University of Granada, 18071 Granada, Spain; seperalta@ugr.es (S.P.); maen@ugr.es (M.E.M.); adolfina@ugr.es (M.A.R.)

**Keywords:** neuroglobin, hyaluronate nanoparticles, stroke, MCAO, proteomics, neuroprotection

## Abstract

Exogenous neuroprotective protein neuroglobin (Ngb) cannot cross the blood–brain barrier. To overcome this difficulty, we synthesized hyaluronate nanoparticles (NPs), able to deliver Ngb into the brain in an animal model of stroke (MCAO). These NPs effectively reached neurons, and were microscopically identified after 24 h of reperfusion. Compared to MCAO non-treated animals, those treated with Ngb-NPs showed survival rates up to 50% higher, and better neurological scores. Tissue damage improved with the treatment, but no changes in the infarct volume or in the oxidative/nitrosative values were detected. A proteomics approach (*p*-value < 0.02; fold change = 0.05) in the infarcted areas showed a total of 219 proteins that significantly changed their expression after stroke and treatment with Ngb-NPs. Of special interest, are proteins such as FBXO7 and NTRK2, which were downexpressed in stroke, but overexpressed after treatment with Ngb-NPs; and ATX2L, which was overexpressed only under the effect of Ngb. Interestingly, the proteins affected by the treatment with Ngb were involved in mitochondrial function and cell death, endocytosis, protein metabolism, cytoskeletal remodeling, or synaptic function, and in regenerative processes, such as dendritogenesis, neuritogenesis, or sinaptogenesis. Consequently, our pharmaceutical preparation may open new therapeutic scopes for stroke and possibly for other neurodegenerative pathologies.

## 1. Introduction

Stroke has become a global social issue that threatens health, and shortens life expectancy, affecting the quality of life worldwide [1]. Ischemic stroke, the most common type of stroke (80–85%), is caused by a blood clot or by the narrowing of a blood vessel, producing infarcts, generally in the territory irrigated by the middle cerebral artery, affecting the parietal cortex and striatum, which undergo histological and functional damages. In order to assess the severity of the infarct, a combination of magnetic resonance imaging (MRI) and neurological evaluation is used. In fact, the outcomes of imaging and pathological parameters are correlated to the results of learning and memory/mood studies [2].

Both core and penumbra zones can be distinguished within the infarcted brain, the last one being susceptible to recuperation only in cases of mild infarct severity, and when specific treatments are available. In this sense, fast and accurate diagnosis methods are required in order to minimize the functional damage. Moreover, further treatments, such as thrombectomy and/or thrombolysis, are necessary. However, these therapies must be applied within a very narrow therapeutic window after the onset of the stroke, and both have strict secondary effects and entail many risks [3,4,5]. Therefore, when the insult is not treated immediately, the nervous cells of the infarcted area suffer severe damages due to oxygen and glucose deprivation, thus undergoing a number of changes, which include the inhibition of the electron transport chain, enhanced formation of reactive oxygen and nitrogen species, mitochondrial damage, energy depletion, and loss of ionic homeostasis, among others [6,7]. These changes lead to cell swelling, membrane rupture, and eventually neuronal death, as usually occurs in the ischemic core [8]. This neuronal deterioration and death imply the retraction and/or rupture of axons with loss of the corresponding neuronal circuits that, if not replaced, lead to functional damage that is difficult to recover from [9].

Consequently, any action against stroke, both prophylactic and therapeutic, should be addressed not only to restore blood circulation in the affected area, but also to boost the mechanisms that prevent cell and tissue damage. Therefore, controlling the production of free radicals and oxidative damage, curbing the unleashing of neuronal death (that induce inflammatory signals), and even activating the processes of neurogenesis and synaptogenesis would be possible therapeutic targets to be explored. In the last decades, new therapies targeting the restorative processes mentioned have been developed, but with disappointing outcomes [10,11].

Accumulating evidence has clearly demonstrated that the oxygen-binding protein Ngb [12] is an endogenous neuroprotective molecule in numerous neurological diseases, and particularly against hypoxic/ischemic (H/I) and oxidative stress-related insults [13]. Its neuroprotective activity has been described in numerous publications from cultured neurons to animal models [14,15,16,17,18,19], although the molecular mechanisms on which Ngb bases its neuroprotective ability are just beginning to be investigated [20]. Specifically, it is known that Ngb interacts with proteins involved in signal transduction and regulation, such as PI3K or AMP-activated protein kinase (AMPK), both involved in mitochondrial functions and cell metabolism [21]. In fact, Ngb plays a central role in mitochondria by promoting the overexpression of antioxidant enzymes through the induction of Nrf2 [22]. Other molecular pathways, such as PTEN/PI3K/Akt or Wnt pathways, related to neurite outgrowth and neurogenesis, respectively, have also been reported to be regulated by Ngb [23,24].

On the other hand, it is known that the expression of Ngb is increased in acute cerebral H/I in murine models [25], and even in humans after stroke [26], indicating that Ngb is very sensitive to H/I. Interestingly, its protective role has been questioned when Ngb is expressed at the physiological level [27,28]. In fact, different studies indicate that Ngb only offers real protection after ischemia when it is overexpressed [29]. In this sense, Ngb-overexpressing transgenic (Ngb-Tg) mice have been used to study the neuroprotective role of Ngb, not only after stroke, but also in other neurological disorders. Specifically, Shang and collaborators [30] demonstrated that overexpressing Ngb exerts significant neuroprotective effects after mechanical injury. In the same direction, in a model of Ngb-overexpressing transgenic mice, Khan and collaborators [31] showed a reduction by 30% in the cerebral infarct size after MCAO; similar results in this same model were found by Raida and collaborators [29] 24 h after ischemia, and in an ischemic preconditioning model of MCAO by Liu and collaborators [16]. Moreover, the reduction of brain infarction in Ngb-Tg mice was sustained up to 14 days after ischemia compared with wild type controls [32]. On the other hand, knocking-down Ngb expression increases neuronal hypoxic injury in vitro, and ischemic injury in in vivo models [32,33].

The above-mentioned studies underlie the important role of Ngb in nervous tissue repair, and how its overexpression is neuroprotective against transient focal cerebral ischemia [34]. Thus, it would be essential to increase the Ngb levels in the infarcted area in order to exploit the neuroprotective effects of Ngb after stroke [34,35]. An adequate approach to accomplish this goal could consist of the direct pharmacological administration of Ngb; however, its molecular size and conformation hamper its penetration through the blood-brain barrier (BBB) [36,37]. Certainly, the BBB can be disrupted transiently [38,39] or continuously [40] after stroke, and during the acute injury phase. In this regard, Ngb could be able to easily reach the central nervous system after a stroke. Notwithstanding, Cai and collaborators [41] reported that this protein couldn’t efficiently cross the BBB in ischemic mice, not even during the acute phase of the stroke. Consequently, the administration of systemic Ngb has been a more than questionable therapeutic strategy in stroke [11,14]. A way to overcome this problem is using cell-penetrating peptides or viral vectors [41,42]. However, this approach showed several disadvantages [43], and we have not found new attempts to use them to deliver Ngb to the brain since the aforementioned publications. An alternative option would be the binding of Ngb to nanocarriers that, beyond its demonstrated physiological feasibility and compatibility, could be susceptible to labelling that may help in monitoring its specific targets [44]. In fact, these nanocarriers can transport large amounts of proteins, and easily cross biological membranes, increasing the bioavailability of the cargo, and protecting it from enzymatic degradation [45,46]. In this context, our group designed polymeric nanoparticles of sodium hyaluronate coated with chitosan and glycerol tripalmitin that satisfactorily crossed the BBB, and were efficiently endocytosed by the nervous cells [47]. Further and beyond, we constructed NPs loaded with recombinant Ngb that have successfully delivered Ngb into the brain parenchyma. This formulation was administered immediately after stroke, and reached the damaged cerebral parenchyma at early stages, remaining at least up to 24 h after the onset of reperfusion [48].

Based on this background, the current research has been addressed to demonstrate that Ngb attached to hyaluronate-NPs (Ngb-NPs) may exert a neuroprotective role in stroke by influencing different biological processes. To achieve this objective, we propose to study the biological implications of our pharmaceutical preparation in rats submitted to MCAO, an animal model of stroke. More specifically, we aim to analyze the survival rates and neurological outcomes of the stroke animals; to perform MRI and histological studies of the brains, as well as an assessment of the oxidative/nitrosative stresses of the infarcted brain area with or without Ngb administration. Finally, in order to deepen the molecular mechanisms by which Ngb exerts its neuroprotective action, proteomic approaches are used to investigate changes in protein expression induced by both stroke and Ngb treatment.

## 2. Results

### 2.1. Ngb Improves the Survival and the Neurological Outcomes of the MCAO-Ngb Animals

**Figure 1:**
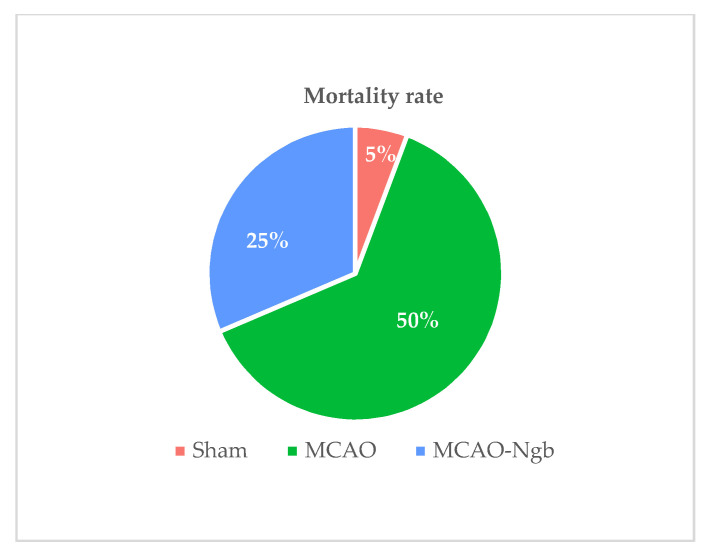
Mortality rates of sham (*n* = 23), MCAO (*n* = 44), and MCAO-Ngb (*n* = 27) groups after 24 h of reperfusion.

Figure 1 shows the mortality rates in the three experimental groups 24 h after surgery. As shown, the rates found in MCAO-Ngb animals fell down to half (25%) in relation to the MCAO group (50%).

**Figure 2:**
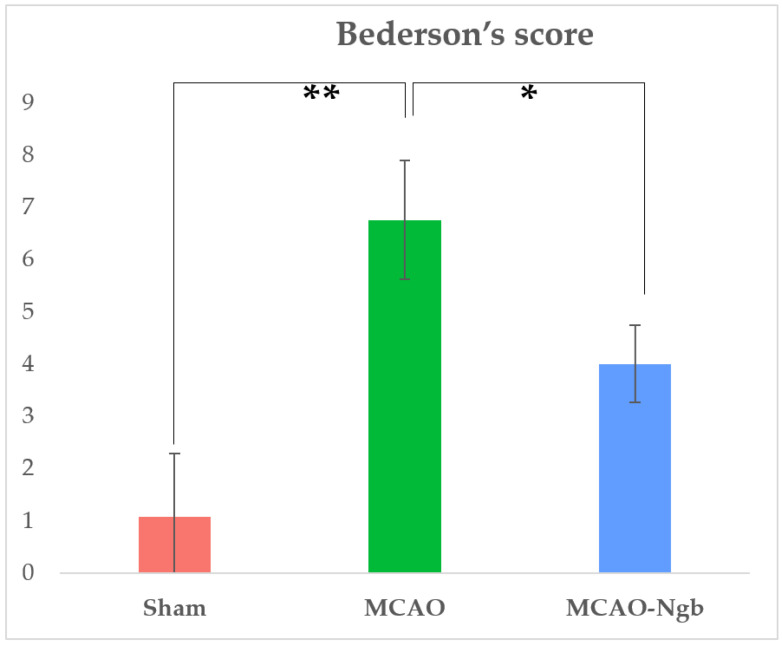
Results of Bederson’s score performed in sham (*n* = 22), MCAO (*n* = 22), and MCAO-Ngb (*n* = 20) groups after 24 h of reperfusion, and immediately before sacrifice. Data are average values of five experimental animals in each group (* *p* < 0.05; ** *p* < 0.01).

Moreover, MCAO animals treated with Ngb (MCAO-Ngb) also improved their neurological outcomes, with a score of 4 points in relation to the non-treated group (MCAO), which reached near to 7 points in Bederson’s scale (Figure 2).

### 2.2. Ngb-NPs Cross the BBB, and are Endocytosed by Neurons

Immunofluorescence techniques and further confocal microscopy observations were performed to ascertain the penetration of the Ngb-NPs into the brain parenchyma, but mainly to determine its cellular localization at the site of the lesion, the parietal cortex, in MCAO animals 24 h after the onset of reperfusion.

**Figure 3:**
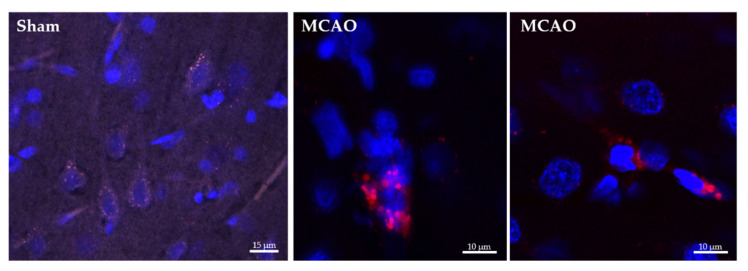
Confocal microscopy images of histological sections of the infarcted zone from sham (**left**) and MCAO (**middle** and **right**) animals taken 24 h after the systemic injection of the hyaluronate NPs. Several nervous cells, whose nuclei are stained in blue with DAPI, show empty NPs grouped as cytoplasmic red vesicles due to its labelling with rhodamine fluorescent dye.

**Figure 4:**
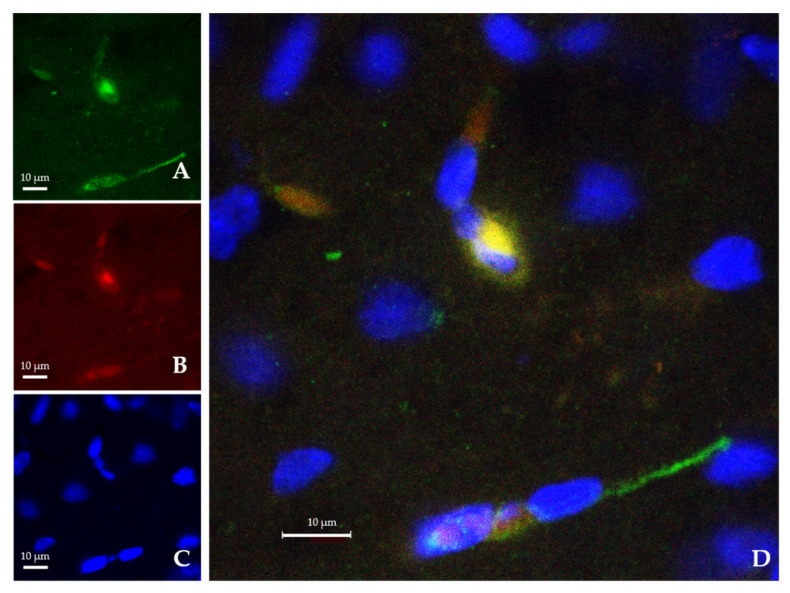
Confocal microscopy images from the infarcted zone of MCAO animals taken 24 h after the systemic injection of the Ngb-NPs. (**A**) Cy2 green fluorescence represents Ngb. (**B**) Rhodamine red fluorescence detects NPs. (**C**) DAPI blue fluorescence marks cell nuclei. (**D**) Merge image showing the colocalization of Ngb and NPs inside the nervous cells. Ngb attached to NPs appears in yellow, due to green and red merge.

**Figure 5:**
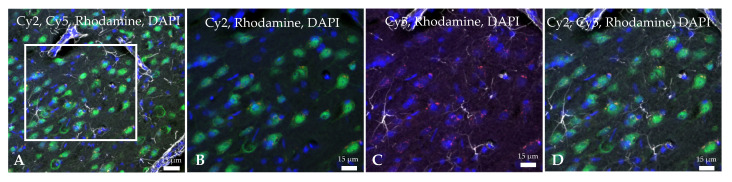
Confocal microscopy images of the infarcted zone from a MCAO-Ngb animal after 24 h of reperfusion. Rhodamine red fluorescence detects the Ngb-NPs, whereas Cy2 green fluorescence binds to NeuN (neurons): Ngb attached to NPs appears in yellow, due to green and red merge. Cy5 dye bound to GFAP (astrocytes) is digitally shown in grey, and DAPI dye marks cell nuclei in blue. (**A**) Merge image showing neurons (NeuN) stained in green with yellow cytoplasmatic vesicles containing Ngb-NPs. Astrocytes are shown in grey. Only few red Ngb-NPs not associated with neurons are observed. (**B**–**D**) Higher magnification of the zone delimited by the white square in image (**A**).

Both empty-NPs (Figure 3) and Ngb-NPs (Figure 4) crossed the BBB, reached the brain parenchyma, and were endocytosed by the nervous cells. In addition, we have identified the NPs inside the neurons of the infarcted zone (Figure 5). As shown in Figure 5 (A and D), most of the Ngb-NPs appeared inside the neurons as yellow granules in the overlaid confocal images, due the colocalization of red rhodamine-marked NPs with NeuN labelled with Cy-2 green fluorescence. As observed after 24 h of reperfusion, only few NPs were located outside the neurons. Cy5 fluorescence, digitally shown in grey, identified GFAP (astrocytes), which barely showed NPs at this reperfusion time.

### 2.3. Ngb-NPs Treatment does not Affect the Infarct Size or the Oxidative/Nitrosative Stresses, but Improves the Histological Outcomes

**Figure 6:**
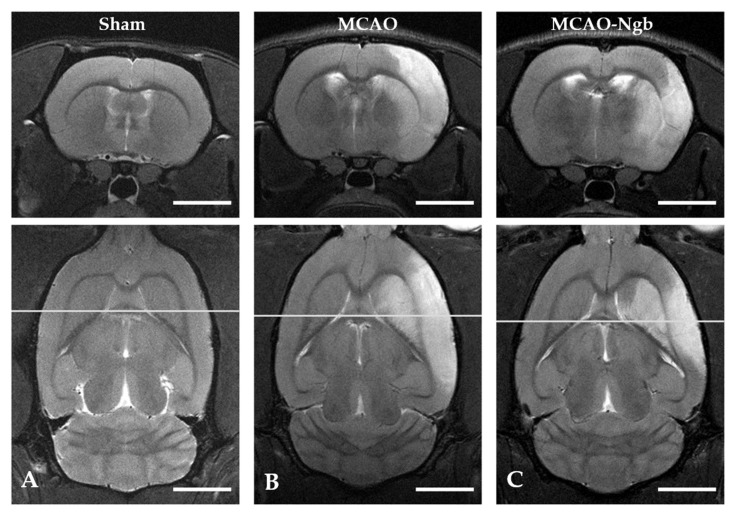
Cerebral T2 axial and coronal neuroimages of sham, MCAO, and MCAO-Ngb animals taken after 24 h of reperfusion. The infarcted area is visible in images (**B**,**C**) in white, but not in (**A**). Scale bars: 5 mm.

**Figure 7:**
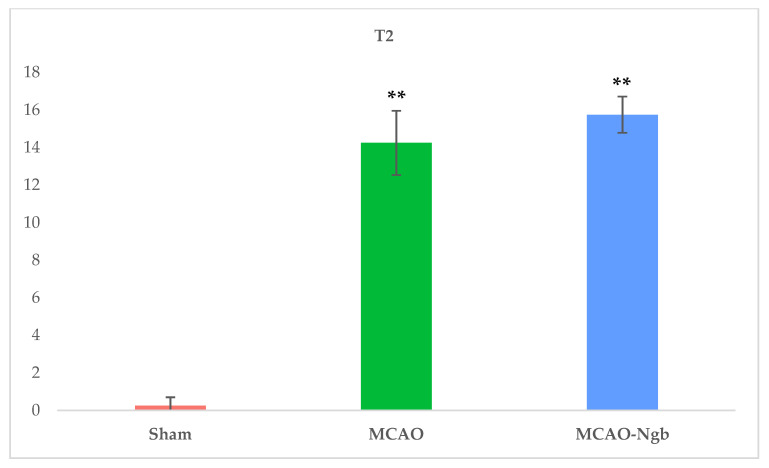
Mean values of the volume of the infarcts in sham, MCAO, and MCAO-Ngb groups calculated from the T2 neuroimages. Data have been calculated using ImageJ software, and are expressed in arbitrary units. Data are average values of six experimental animals in each group (** *p* < 0.01).

Figure 6 shows MRI T2 representative images acquired 24 h after the MCAO surgery. The T2-weighted MR axial and coronal neuroimages of the animals submitted to MCAO showed no changes in the infarct size between MCAO and MCAO-Ngb groups (Figure 7).

**Figure 8:**
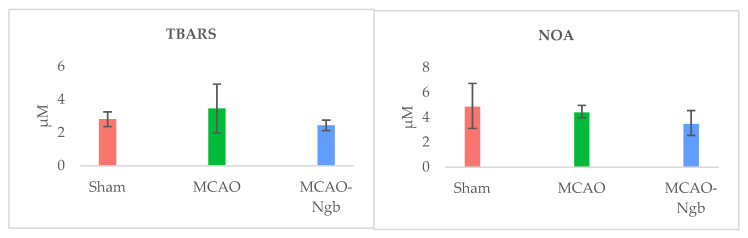
Determinations of oxidative (TBARS) and nitrosative (NOA) stresses in sham, MCAO, and MCAO-Ngb animals. Data are average values of five experimental animals in each group. No significative differences are found.

The determination of the oxidative (TBARS) and nitrosative stress (NOx) in the tissues of MCAO and MCAO-Ngb animals also showed non-significant changes in relation to sham animals (Figure 8).

**Figure 9:**
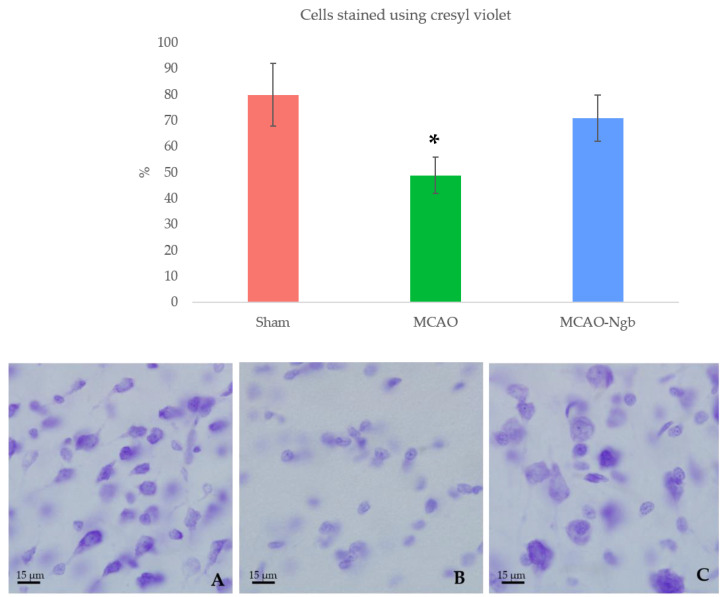
Upper panel: percentage of cells stained by cresyl-violet for each group: sham (**A**), MCAO (**B**), and MCAO-Ngb (**C**), measured using ImageJ. Animals from MCAO group show fewer neurons than rats from sham and MCAO-Ngb groups. Data are average values of 5 repetitions in 10 sections of 5 experimental animals in each group (* *p* < 0.05). Lower panel: representative microphotographs of the infarct zone (parietal cortex) of animals stained with cresyl-violet.

Nevertheless, the microscopic analysis of the histological sections from the damaged area stained with cresyl-violet showed that MCAO-Ngb individuals exhibited less histological damage than the MCAO animals, where scarce cell bodies could be found (Figure 9).

### 2.4. Proteomic Findings Reveal Potential Biological Processes Involved in the Protective Action of Ngb

#### 2.4.1. Protein Identification and Quantification

The total amount of proteins initially identified and quantified in the 12 samples studied was of 4077 proteins (expressed by MaxQuant as “Protein groups”). This initial set of proteins underwent several steps of filtering using the Bioconductor package DEP, as previously explained. The statistical analysis performed using the DEP package provides an integrated analysis workflow for robust and reproducible analysis of mass spectrometry proteomics data for differential protein expression or differential enrichment. Specifically, the 4077 proteins obtained (after the MaxQuant analysis) became 3984 when contaminants and reverse (decoy) sequences were removed. Then, proteins with only one peptide were also filtered, obtaining 3298 proteins. Finally, this number became 2573 (Supplementary data: Differential analysis results annotated), considering only those proteins present in at least one sample in each of the three studied conditions, grouped as sham, MCAO, and MCAO-Ngb.

#### 2.4.2. Quality Control and Differential Analysis

**Figure 10:**
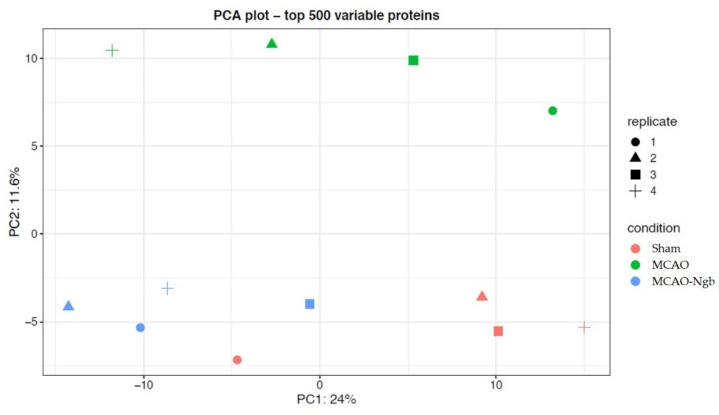
Quality control plot of the two first principal components (PCA) using the top 500 variable proteins among the three groups (sham, MCAO, and MCAO-Ngb). As shown, MCAO-Ngb animals were clustered closer to the sham control group than to the MCAO non-treated group.

A first approach for quality control, based on filtering the proteins found in all samples from the three groups, quantified almost 2000 proteins, of which the 500 most variable were chosen to perform a principal components analysis (PCA). As shown in Figure 10, the PCA grouped the different samples according to their corresponding condition, i.e., sham, MCAO, or MCAO-Ngb groups; interestingly, the MCAO-Ngb animals were clustered closer to the sham control group than to the MCAO group.

**Figure 11:**
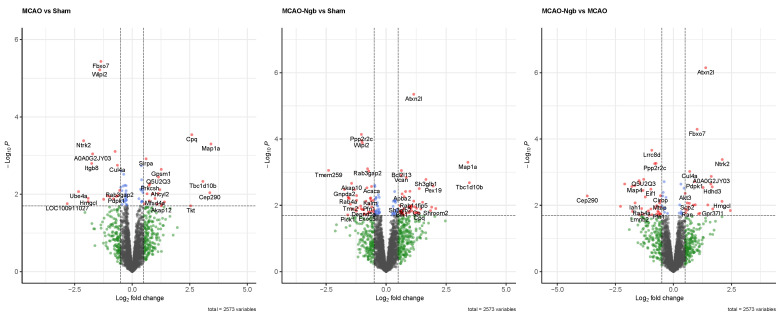
Volcano plots for the three comparisons using *p* < 0.02 and Log2 fold change of 0.5 as threshold. When comparing MCAO vs. sham, proteins FBXO7, WIPI2, NTRK2, A0A0G2JY03, and ITGB8 are underexpressed, whereas MAP1a and CPQ are overexpressed. In the comparison between MCAO-Ngb and sham, proteins PPP2r2c, WIPI2, and LRRC8d are shown to be underexpressed, whereas ATXN2l, MAP1a, and TBC1d10b are overexpressed. The comparison between MCAO-Ngb and MCAO involves the over expression of ATXN2l, FBXO7, and NTRK2.

A second approach for quality control was performed under a *p*-value of 0.02 and a Log2 Fold change of 0.5 as cut-offs. These thresholds are reliable enough [49] to identify the most significant changes in the expression of proteins in our model induced by the stroke and the treatment with Ngb. Figure 11 shows the volcano plots obtained in each of the three comparisons included in the study, where several proteins presented a clear over- or underexpression depending on stroke and/or treatment with Ngb. Specifically, when comparing MCAO vs. sham, proteins FBXO7, WIPI2, NTRK2, A0A0G2JY03, and ITGB8 appeared clearly underexpressed, whereas MAP1a and CPQ were overexpressed, indicating a clear effect of the stroke (MCAO injury) on the individuals. In the second comparison, MCAO-Ngb vs. sham, proteins PPP2r2c, WIPI2, and LRRC8d were underexpressed, whereas ATXN2l, MAP1a, and TBC1d10b appeared overexpressed. Finally, in the comparison between MCAO-Ngb and MCAO, which demonstrates the effects of the treatment with Ngb-NPs in animals submitted to stroke, the most evident changes involved overexpression of ATXN2l, FBXO7, and NTRK2. These results show that the treatment with Ngb-NPs induces expression of new proteins, or even reverses the expression induced by the effect of the stroke.

#### 2.4.3. Hierarchical Clustering

**Figure 12:**
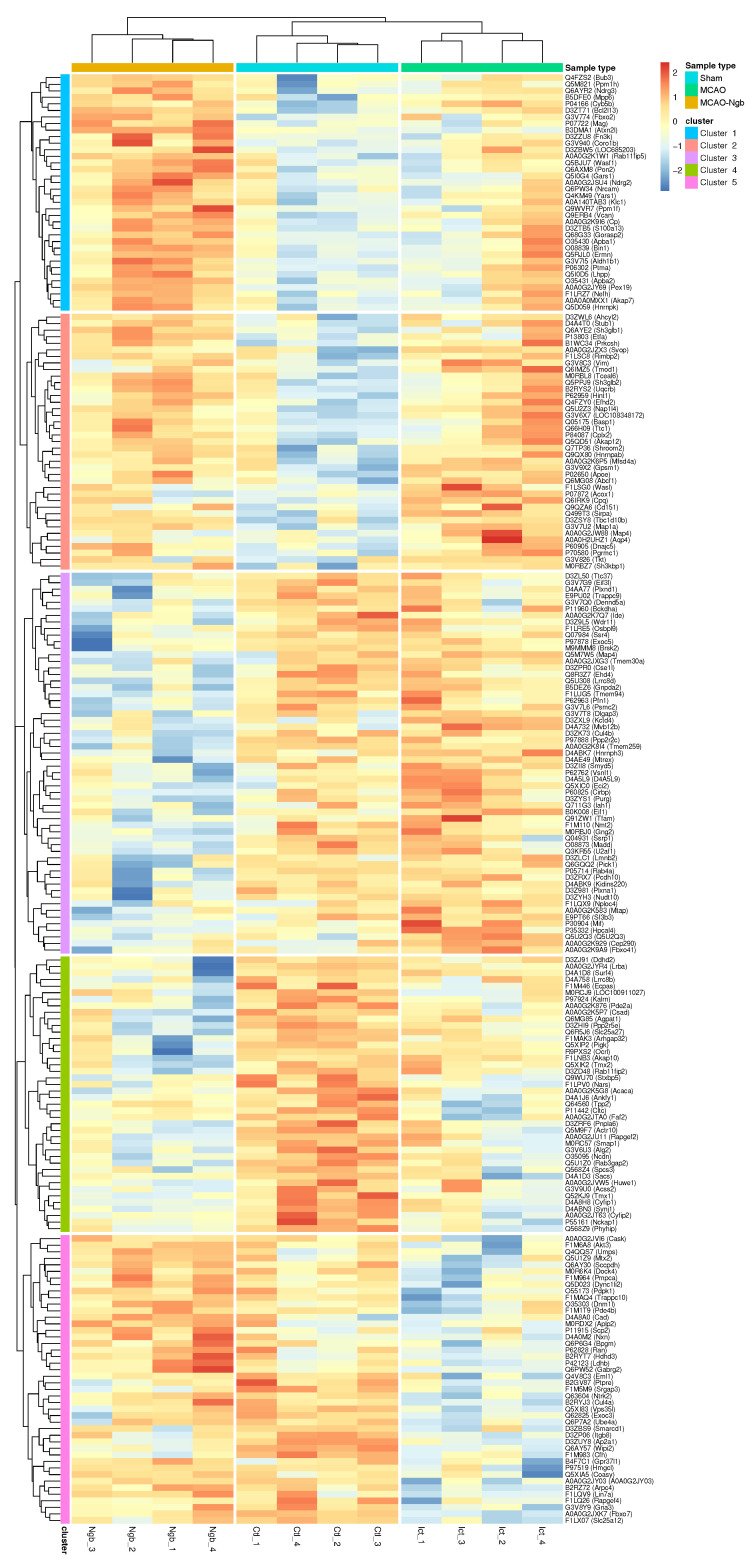
Hierarchical clustering using the intensities of proteins that present a *p* < 0.02 in at least one of the three comparisons (MCAO vs. sham, MCAO-Ngb vs. MCAO, and MCAO-Ngb vs. sham). The five main aggrupation of proteins are highlighted as cluster 1 to cluster 5 at row level. The three types of samples (sham, MCAO, and MCAO-Ngb) are highlighted at column level.

A hierarchical clustering study has been performed using proteins that showed expression changes in at least one of the comparisons among the three groups under a *p*-value lower than 0.02. This filter produced a set of 219 proteins affected. For each protein and sample, the normalized intensity was used to make a matrix of 219 × 12. The matrix was scaled obtaining a maximum of +2.42, and a minimum of −2.86. This matrix was used to perform a hierarchical cluster using the pheatmap package, where both rows (proteins) and columns (samples) have been clustered, i.e., they have freely organized themselves using the individual intensities of the matrix (unsupervised machine learning both for proteins and samples). The optimal number of clusters has been obtained using the find_k function from the dendextend R package. The proteins were grouped in five clusters that accurately describe how protein intensities have been organized in different categories that can be represented in a hierarchical cluster (Figure 12). In this representation, both proteins and genes have been used to annotate the rows; the columns are represented by the 12 samples used in the study.

The three conditions: MCAO-Ngb, sham, and MCAO (in this order) have been used to annotate the samples, whereas the five main clusters are shown as cluster 1 to cluster 5. Different general patterns of intensities are found for each of the clusters and sample types (activation is represented in red, and underexpression in blue). Once again, it is clear that the different expression intensities of these 219 proteins are able to perfectly classify the 12 samples into the three conditions (groups) of our study. The hierarchical cluster also shows the trends of the changes in the protein expression for the five clusters (1 to 5) in the MCAO-Ngb, sham, and MCAO groups, respectively. Samples belonging to the group of the stroke condition (MCAO) show, in general, more variability than the other two conditions (sham and MCAO-Ngb), especially in cluster 1 and cluster 4, which could point to a high heterogeneity in the biological reaction to a stroke event.

#### 2.4.4. GO and Pathways Enrichment

Two enrichment studies have been performed. First, each of the five clusters of the hierarchical cluster study was analyzed separately. Second, all the proteins were analyzed together (the proteins in this study included 517 proteins with a *p*-value lower than 0.05 in at least one of the three comparisons); the enrichment was performed with STRING database, version 11.0, and using *Rattus norvegicus* species.

#### 2.4.5. Individual Enrichments in Each of the Five Clusters

Five different experiments of enrichment, using gene ontology (GO), biological function category (Gene Ontology Resource), and KEGG pathways (KEGG PATHWAY Database) have been performed using the proteins in each of the clusters shown in the hierarchical cluster (Figure 12). Results are displayed in Table 1, where, as exposed, only clusters 1 to 4 showed remarkable results.

Actin binding has been found as one of the biological functions most affected, as shown in clusters 1 and 2, with three and four genes involved, respectively. One of these genes in cluster 2 corresponds to Map1a, previously found as overexpressed due to stroke. Another biological function affected is “GTPase binding”, grouped in cluster 3 with five genes involved; and “Ras GTPase binding” in cluster 3 and 4 with four genes involved in both clusters. Special attention should be given to the KEGG pathways “endocytosis” and “protein processing in endoplasmic reticulum”, both concerning cluster 2, and showing a total of four and three dysregulated genes, respectively.

#### 2.4.6. Global Enrichment Using Differentially Expressed Proteins

A GO enrichment (only for biological function) and pathway enrichment (for KEGG pathways) have been performed using the 517 proteins that showed a *p*-value lower than 0.05 in at least one of the three comparisons performed (MCAO vs. sham; MCAO-Ngb vs. sham; MCAO-Ngb vs. MCAO). The results can be found in Table 2. A total of 113 terms show some enrichment (FDR<0.05); 75 of them are biological function terms, and the other 37 are KEGG pathways. Terms with a background (complete set of genes in the category) higher than 300 have been removed from this list. The row corresponding to the KEGG pathway “endocytosis”, with 28 proteins mapped, has been split. This global enrichment endorses the results obtained in the individual enrichment previously performed. Thus, the most altered biological functions are related with cytoskeletal protein interactions (i.e., “actin” or “microtubule bindings”), and “GTP signaling” and “energy metabolism” (i.e., GTPase, GTP, GDP, or Ras GTPase bindings). In relation to KEGG pathways, the most significant ones were “endocytosis”, as well as “protein synthesis, processing and degradation”.

#### 2.4.7. Pathway Analysis

**Figure 13:**
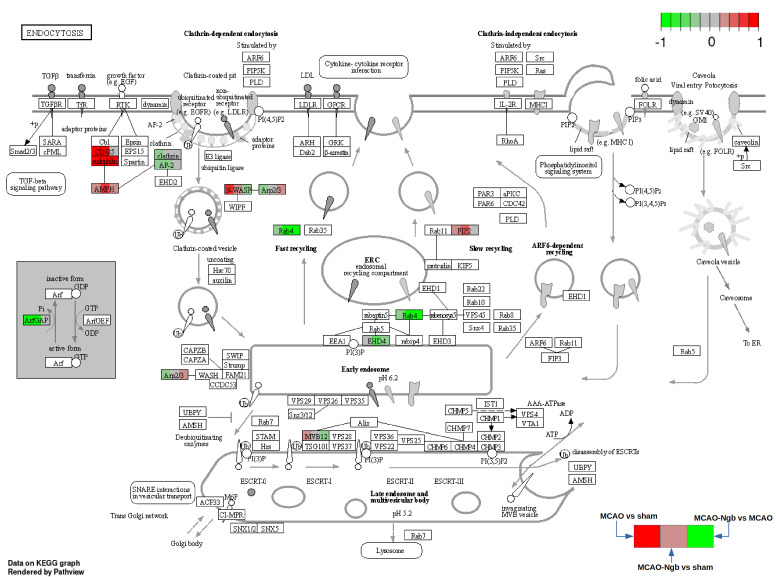
KEGG pathway hsa04144: endocytosis. Arrows and symbols explanation: https://www.genome.jp/kegg/document/help_pathway.html (accessed on 25 November 2021).

The pathway analysis using Kegg pathways has been implemented with the Bioconductor package Pathview (pathview). Only proteins with a *p*-value lower than 0.05 in at least one of the three comparisons have been used. The pathways selected are those that have shown significant enrichment in the previous section (Table 2), and showed some relationship with the matter under study. Figure 13 shows the “endocytosis pathway” as an example of one the most affected pathways, where each protein area is divided into three sectors: the one to the right shows the fold change for the MCAO vs. sham, the one in the center for the MCAO-Ngb vs. sham, and the one in the right for the MCAO-Ngb vs. MCAO. Red color means overexpression, and green means underexpression.

This last analysis ratifies that the treatment with Ngb-NPs is involved in some transcendent biological processes, such as endocytosis, protein metabolism, synaptic function, and neurotransmission (synthesis of neurotransmitters—GABA, serotonin, glutamic acid—or synaptic vesicles traffic), or even in molecular pathways recurrent in some neurodegenerative diseases, such as Huntington’s, Parkinson’s, or Alzheimer’s.

## 3. Discussion

The present research demonstrates that, when injected systemically, Ngb-NPs can cross the BBB, and be endocytosed by neurons of the infarcted area in a rat model of stroke (MCAO). Once there, Ngb exerts its neuroprotective action only after 24 h of reperfusion. It has been proved that the animals submitted to stroke, and treated with Ngb-NPs have higher survival rates and better behavioral scores than the MCAO animals. The histological status also improved with the treatment, although at this early time of reperfusion, no changes neither in the infarct volume nor in the oxidative or nitrosative stress assessments were detected in the damaged infarcted area. Finally, the proteomic changes induced by stroke and the Ngb-NPs treatment has been analyzed with the aim of unravelling the molecular pathways by which Ngb exerts its neuroprotective action. These changes affect some biological processes, such as endocytosis, cytoskeletal remodeling, or some metabolic routes, all of them related to damage, and restorative mechanisms that are involved in dendritogenesis, neuritogenesis, or sinaptogenesis.

Currently, there are multiple restorative therapies against stroke addressed to avoid some processes of the ischemic cascade, such as oxidative/nitrosative stresses, or glutamate excitotoxicity or Ca^2+^ overload, which may finally may trigger the activation of necrosis and apoptosis processes [50]. These therapies range from the most conventional (anticoagulants, decompressive surgery, or hypothermia) to the most recent (monoclonal antibodies, cell-based therapy, or even robotics stimulation) [51,52]. Additionally, pharmacological treatments using growth factors (BDNF, EGF plus EPO, and hCG plus EPO), specific neurotransmitters (oxalate, glutamate), or nitric oxide (NO) synthase inhibitors have been tested [53,54,55]. Interestingly, the administration of nutritional supplements in the diet is gradually reaching greater interest [56,57]. Other new promising therapies are based on emergent neuroprotectants, such as antioxidant enzymes like Peroxiredoxin-6 [58], or even other proteins with a wide neuroprotective capacity, such as the protein Ngb [13]. However, the therapeutic usage of this protein is still limited because of its difficulty to cross the BBB. To avoid this handicap, different types of drug delivery systems have been developed, such as the use of cell-penetrating peptides (CPP). In this vein, we have synthesized different types of hyaluronate nanoparticles optimizing their size and electrical charge, making them suitable to avoid the immune system, and cross the BBB after stroke [47]. Then, we selected the best typology of these NPs to transport an adequate dose of rat recombinant Ngb from the bloodstream to the cerebral parenchyma. This new pharmaceutical preparation was injected immediately after the stroke, and was delivered into the ischemic cerebral area as early as 2 h after stroke, and was still detectable after 24 h of reperfusion [48]. In order to ascertain the biological implications and neuroprotectant ability of our pharmaceutical preparation of Ngb, our first objective here was to demonstrate that the Ngb-NPs could be endocytosed by neurons. This approach has been confirmed using immunofluorescence and confocal microscopy; in fact, the confocal images obtained have allowed us to assert that Ngb-NPs have not only crossed the BBB, but have finally been captured by neurons, as demonstrated with the colocalization of such Ngb-NPs with the specific neuronal protein, NeuN [59]. Actually, we do not know the exact pathway followed by the Ngb-NPs from blood to neurons, and although it has been described that the BBB can be disrupted transiently [38,39] or continuously [40] during stroke, some authors have also reported that Ngb does not efficiently cross the BBB in ischemic mice [41]. Therefore, the Ngb-NP should go through the many cellular layers of the BBB by transcytosis mechanisms until they reach the neurons. Our microscopic images show that, after 24 h of reperfusion, the neurons have already captured most of the NPs systemically injected in the vascular network; these NPs appear clustered in their cytoplasm, although a few of them are also located within other nervous cells. Hence, the specific cytoskeletal protein of astrocytes GFAP barely shows colocalization with our NPs. Interestingly, it has been reported that isolated cultured astrocytes express and release endogen Ngb towards neurons packed inside of exosomes [60]. Considering the above, it would be possible to speculate that astrocytes from the BBB could release our NPs using this same pathway, but also, this transcytosis would be over after 24 h of the onset of the reperfusion.

Another important subject of our research concerns the fact that the MCAO-Ngb animals doubled their survival rates, and showed improved neurologic scores compared to non-treated animals. There are many reports that document the protective role of Ngb in stroke and other neurodegenerative diseases [13], but only a few of them have analyzed survival rates and/or neurological outcomes after stroke. Xue and collaborators [61] found that high serum levels of endogen Ngb, whose peak is reached 72 h after ischemic stroke, correlated significantly with both infarct size and admission NIHSS score in humans. A study that combined overexpression of Ngb and an inhibitor of the c-jun N-terminal kinase (JNK), carried out in male spontaneously hypertensive prone stroke rats, reported that this treatment reduced the infarct size, and improved the neurologic outcome 14 days after transient MCAO (tMCAO) [62]. Our research shows that as early as 24 h after stroke, the survival rates and the neurological scores improved with the Ngb-NPs treatment; on the other hand, no changes were detected in the infarct size, or in the oxidative and nitrosative stresses statuses. Thus, we hypothesize that 24 h of reperfusion may be too soon to observe these types of changes, despite the findings of better outcomes in the tissue status. Certainly, new studies with longer reperfusion times and even higher Ngb dosage should be conducted for a better assessment of the therapeutic effects of Ngb.

Regarding the most significant proteomic changes induced by Ngb-NPs in stroke animals, they involved proteins featuring some key pathways, such as endocytosis and vesicular traffic, cytoskeletal remodeling, metabolism, or synaptic transmission. All these pathways are highly related to neurodegenerative tissue damage and regeneration. Interestingly, a total of at least 219 proteins from these pathways underwent noticeable expression changes, although we have focused on the most significant proteins, previously identified in the volcano plots. Particularly, the ischemic lesion induced the underexpression of FBXO7 and NTRK2, although when stroke animals were treated with Ngb-NPs, both proteins underwent a strong recovery and even overexpression. FBXO7 (F-box only protein 7) corresponds to an E3-ubiquitin-protein ligase that participates in the proteasomal degradation of target proteins involved in mitophagy and negative regulation of stress cellular death. At the moment, FBXO7 has been related to Parkinson’s disease [63], although to the best of our knowledge, it has not been directly involved in stroke. In fact, FBXO7 mediates the proteasomal degradation of UXT-V2, causing the inhibition of the NF-κB signaling pathway, suggesting that FBXO7 plays roles in mitochondrial transport and Wnt signaling regulation [64], a pathway highly related to neurogenesis [24]. NTRK2 (high affinity nerve growth factor receptor) is a receptor of BDNF that is also involved in neurogenesis and neuronal plasticity; this protein has been related to stroke throughout the pathway BDNF-TrKBPI3K/Akt. Therefore, Ngb could activate the PI3K/Akt pathway by increasing the BDNF levels, and thus, enhance synaptic plasticity, and repair processes of damaged neurons [65].

Other proteins, such as MAP1a and CPQ, are overexpressed due to MCAO lesions, and remain elevated even after the treatment with Ngb-NPs. MAP1a (microtubule-associated protein 1a) is a structural protein mainly located in the dendrites of adult neurons involved in the filamentous cross-bridging between microtubules and other skeletal elements. Thus, this protein controls the microtubule assembly [66], but can also interact with other cellular components, including filamentous actin and signaling proteins [67]. Interestingly, the activity of MAP1a is controlled by upstream signaling mechanisms through the mitogen-activated protein (MAP) kinase, suggesting that its dendritic remodeling activity depends on this pathway. The fact that the treatment with Ngb-NPs maintains MAP1a levels elevated after induction by stroke, is probably to favor its restorative activity repairing the dendrites of neurons damaged by the ischemic stroke. Regarding CPQ, a carboxypeptidase that may play an important role in the hydrolysis of circulating peptides, it also follows the same expression pattern as MAP1a. To our knowledge, it has not been previously detected in a stroke-damaged brain, but our results document that MCAO induces its expression in both MCAO and MCAO-Ngb animals.

Another downregulated protein is WIPI2. This protein (WD repeat domain phosphoinositide-interacting protein 2) is involved in autophagy during mitosis, via CUL4-RING ubiquitin ligases-mediated WIPI2 polyubiquitination and proteasomal degradation [68]. In fact, WIPI2 protein level, regulated by MTORC1 and HUWE1 (an E3 ubiquitin ligase), plays a determinant role in cellular autophagy intensity, suggesting that the quantity control exerted by WIPI2 affects autophagy-related physiopathological processes [69]. Our results showed a decrease of this protein after stroke, remaining underexpressed with Ngb treatment; consequently, stroke could regulate the intensity of autophagy throughout the underexpression of this protein.

A specific feature of the Ngb-NPs treatment is the overexpression of ATX2L. This protein is involved in the assembly of the stress granules (formed in response to diverse types of stresses), and in the cytoplasmic mRNA processing bodies [70]. Moreover, this protein contributes to the cytoskeletal reorganization, and to the recovery of the mitochondrial function through its role in different metabolic pathways. In fact, this protein has also been related to the “Wnt signaling pathway” [71], which, as was previously mentioned, is involved in neurogenesis.

On the other hand, our proteomic approaches also show important expression changes in up to 28 different proteins involved in endocytosis, predicting that stroke and the later treatment with Ngb may interfere with the vesicular traffic. One of these proteins is the Ras-related protein RAB-4A, which is underexpressed, but only due to Ngb-NPs treatment. This is a small GTPase associated-protein with endocytic compartments and a key regulator of early endosomes recycling [72]. In addition, it cycles between an active GTP-bound and an inactive GDP-bound state, being associated with endosomal vesicular traffic through “VEGFR2 signaling”, a pathway related to angiogenesis [73]. What is more, this pathway is associated with the protein, endophilin (ENDO), which is overexpressed either in MCAO and MCAO-Ngb animals. Endophilin was identified as a component of clathrin-mediated endocytosis [74], and its deficiency reduces VEGFR2 internalization, and, therefore, regulates angiogenesis. EH-domain-containing 4 (EHD4) is also a protein of the endocytic network that is downregulated both by stroke and treatment with Ngb. This protein controls early endosome trafficking, promotes the recycling of internalized transmembrane proteins [75], and is related to the pathways underlying the axonal transport as well [76]. Another important protein involved in the endocytic pathway is the neural Wiskott–Aldrich syndrome protein (N-WASP or WASL), an actin nucleation factor responsible for the polymerization of branched actin filaments that underwent upregulation by MCAO, but later downregulation due to the treatment with Ngb. The activity of this protein is essential for myelin membrane wrapping and, therefore, for neuritogenesis [77]. Additionally, N-WASP takes part in the development of dendritic spines and synapses [78].

In the light of the important number of endocytic proteins entailing pathways such as dendritogenesis, neuritogenesis, or even sinaptogenesis, it can be inferred that both the MCAO injury and the treatment with Ngb-NPs are the main inductors of the regulation of these repairing mechanisms. Certainly, the displacement of the NPs (empty or linked to Ngb) from blood towards neurons could also influence the endocytic pathway, but this should have been equally observed in the three groups, as all of them received the same dose of hyaluronate NPs.

In consequence, Ngb seems to induce changes in the expression of a myriad of proteins associated to a variety of biological processes, all of them contributing to reveal the molecular bases of its neuroprotective role, mainly centered in avoiding neuronal death, and triggering reparation mechanisms.

## 4. Materials and Methods

### 4.1. Biosynthesis of Ngb-NPs

By means of ionic gelation methods, we previously designed different types of hyaluronate NPs coated with chitosan and glycerol tripalmitin. Final size (245 nm) and surface charge (about 32 mV) of the NPs were adequate for their use as therapeutic tools. These NP were able to cross through the BBB, and to be endocytosed by nervous cells [47], even after being attached to rat-recombinant Ngb, previously prepared in our lab [48]. These Ngb-NPs were evaluated in size, ζ-potential, encapsulation degree, and in vitro release; in addition, their kinetic liberation was assessed as well. The results showed that the formulation was highly compatible with its pharmaceutical use, and that it could act as a delivery system to transport Ngb throughout the blood towards the brain parenchyma. A comprehensive analysis of the composition and characteristics of these Ngb-NPs has been previously published by our group [47,48].

### 4.2. Experimental Animals

All procedures have been approved by the local Animal Care Committee, and were performed in compliance with the Spanish legislation, and in accordance with the EU Directive 2010/63/EU (2010). The procedures comply with the provisions of current legal regulations and, in particular, with Law 32/2007 amended by Law 6/2013 and by Royal Decree 53/2013. The experimental procedures were carried out in adult (4 months) male Wistar rats (Charles River, Wilmington, MA, USA).

### 4.3. Study Design

The animals (*n* = 94) were homogeneously distributed into three different experimental groups: (1) sham-operated rats injected with empty NPs (sham), used as control; (2) rats submitted to MCAO and injected with empty NPs (MCAO); and (3) rats submitted MCAO and injected with Ngb-NPs (MCAO-Ngb). After surgery, with the mortality rates taken into account (Figure 1), the number of surviving animals used for all analyses and determinations yielded an *n* = 64.

NPs, either empty or bound to Ngb (2.15 mg/mL) [48], were systemically injected via the lateral tail vein immediately after the surgery at the onset of reperfusion. The total number of animals per group was distributed according to each of the techniques listed, minimizing the use of animals, but guaranteeing an adequate “n” per group to ensure an appropriate statistical confidence.

### 4.4. Stroke Model

Transient focal ischemia was induced by occlusion of the middle cerebral artery as previously described [79,80], with slight modifications [47,48]. This model of brain ischemia is considered one of the best models to mimic human ischemic stroke [81], representing an appropriate in vivo procedure to define the protective role of Ngb-NP against stroke.

Briefly, the middle cerebral artery is occluded using a nylon filament suture with a 3–4 mm coating (Doccol Corporation, Redlands, CA, USA), inserted through the right common carotid artery, and advanced until the origin of the middle cerebral artery. A laser-Doppler flow probe (tip diameter 1 mm) attached to a flowmeter (moorVMS-LDF1, Moor Instruments, Axminster, UK) is located over the thinned skull in the MCAO territory (4 mm lateral to bregma) to obtain a continuous measure of relative cerebral brain flow during the surgery. Only animals with a cerebral blood flow reduction over 60% were included in the study. After surgery, anesthesia was discontinued, and rats were returned to their home cages. Animals in the three groups studied were sacrificed 24 h after MCAO procedure.

### 4.5. Mortality Rate and Neurological Outcomes

The mortality rate was assessed by calculating the percentage of animals in each experimental group that survived the MCAO intervention, but died within the 24 h of reperfusion period.

A double-blind evaluation of the neurological outcomes induced by MCAO and reperfusion was carried out in all animals immediately before neuroimaging or sacrifice following Bederson’s neurological scoring [82]. This evaluation was carried out in the animal facilities of the University of Jaén, which are equipped with a specific module for animal behavioral assessment. More specifically, we measured the neurological deficits according to the scoring system depicted in Table 3.

### 4.6. MRI Neuroimage Study

In order to document the extent of the ischemic damage, an MRI was performed prior to euthanasia on a 9.4T Biospec Avance III small animal MR system (Bruker BioSpin, Ettlingen, Germany) equipped with 400 mT/m field gradients, and operated by a Paravision 6.0 software platform. A circular polarized volume resonator was used for signal transmission, and an actively decoupled rat brain quadrature surface coil for signal reception (Bruker BioSpin, Ettlingen, Germany). During the experiment, isoflurane (Abbott Laboratories, Madrid, Spain) was used for anesthesia (3.5% for induction for 2 min, and approximately 1.0 % for maintenance in oxygen at a flow rate of 1–1.5 mL/min). Respiration of the animal was monitored using a pneumatic cushion respiratory monitoring system (Small Animal Instruments Inc., Stony Brook, NY, USA). Animals were placed on a water-heated rat cradle, and the head was immobilized using ear and tooth bars. Body temperature was measured using a rectal thermometer, and maintained at 37 °C. First, fast gradient echo images were acquired as reference images. High resolution T2-weighted MR images were acquired using a RARE (rapid acquisition with relaxation enhancement) sequence with the following parameters: TR = 2500, TE = 33 or 60 ms, in-plane resolution = 83 m, slice thickness = 1 mm, and ETL = 8. T2 parametric maps were also acquired using a multi-echo spin echo sequence (32 echoes and 7 ms inter-echo spacing). The volumes of the infarcts shown in the T2 images were obtained using Image J software. These experiments were performed at the Andalusian Centre for Nanomedicine and Biotechnology (BIONAND).

### 4.7. Histological Studies

Animals were perfused through the left ventricle with pH 7.4, 0.01 M phosphate-buffered saline (PBS), followed by 300 mL of 10% formalin. Brains were removed and post-fixed for a further 4 h in the same fixative at room temperature as previously published (Blanco et al., 2020). Then, the brains were cryoprotected for 48 h by immersion in 0.1 M phosphate buffer (PB) containing 30% sucrose at 4 °C, and embedded in O.C.T. medium. Serial rostro caudal sections (30 μm thick) were obtained using a cryostat (Leica CM1950, Leica Microsistemas S.L.U., L’Hospitalet de Llobregat, Spain). Serial sections were submitted to different histological techniques.

#### 4.7.1. Cresyl-Violet (Nissl) Staining

This technique was used in order to evaluate the histological damage within the brain tissue. Briefly, sections were stained using a 0.1% solution of cresyl-violet, dehydrated, and mounted with DPX.

#### 4.7.2. Immunofluorescence

Brain slices were blocked and permeabilized with 3% normal goat serum and 0.1% PBS-Triton, respectively. They were then incubated overnight at 4º C with non-competent primary antisera in PBS containing 0.2% Triton X-100 in order to avoid crosslink reactions: 1:500 rabbit anti-Ngb (sc-30144, Santa Cruz, Dallas, TX, USA); 1:500 rabbit anti-Glial Fibrillary Acidic Protein (GFAP) (Z0334, Dako Agilent, Santa Clara, CA, USA); and mouse anti-NeuN (neuron-specific protein) (NBP1-92693, Novus, Centennial, CO, USA). After several rinses in PBS, sections were incubated with Cy2-linked goat anti-mouse IgG 1:1000 (PA42002, GE Healthcare, Little Chalfont, UK) or Cy5-linked goat anti-rabbit IgG 1:1000 (PA45004, GE Healthcare, Little Chalfont, UK) correspondingly. After three rinses in PBS, the slices were stained with 4′,6-diamidino-2-phenylindole (DAPI) (124653, Merck, Darmstadt, Germany), 1:2000 for 8 min to visualize the cell nuclei. Then, the sections were quickly dehydrated in graded ethanol series, cleared, and mounted on slides for examination under confocal laser scanning microscope (Leica TCS SP5 II, Leica Microsistemas S.L.U., L’Hospitalet de Llobregat, Spain) in the Central Research Support Services of the University of Jaén (SCAI).

In all the confocal images, rhodamine-stained NPs were visualized in red (excitation 507 nm, emission 525 nm), and the cell nuclei were visualized in blue due to DAPI staining (excitation 358 nm, emission 529 nm). The other markers were visualized with different immunofluorescence dyes conveniently chosen according to the corresponding research purpose. Thus, to show colocalization between NPs and Ngb, Ngb was marked with Cy2 (excitation 492 nm, emission 510 nm), and observed in green. To detect the cellular localization of the NPs in the brain parenchyma, NeuN was marked with green fluorescent-Cy2 (excitation 492 nm, emission 510 nm), and GFAP was marked with Cy5 (excitation 650 nm, emission 670 nm), digitally shown in grey for better understanding.

To ensure that the signal detected in each channel was not due to autofluorescence, TrueVIEW Autofluorescence Quenching Kit (Vector Laboratories, Burlingame, CA, USA) and control samples of rats not injected with NPs were used.

### 4.8. Oxidative and Nitrosative Stresses Determinations

The scavenging properties of Ngb were assessed by determining the oxidative/nitrosative statuses. Lipid peroxidation and NO production were measured using the method described by Buege and Aust [83] (thiobarbituric acid reactive substances, TBARS), and by means of indirect quantification of NO production by measuring nitrate/nitrite and S-nitrose compounds (NOA^™^ 280i, Sievers Instruments Inc.-Zysense, Frederick, CO, USA), respectively, in ischemic brain tissues of the infarcted hemisphere from animals of each experimental group.

### 4.9. Proteomics Analysis

#### 4.9.1. Sample Preparation and Mass Spectrometry Analysis

A total of 12 samples, 4 per condition (sham, MCAO, and MCAO-Ngb), were included in the proteomic studies. First, samples were incubated with 7 M urea, 2 M Thiourea, 4% CHAPS, and 5 mM DTT. Proteins were extracted using a Precellys tissue homogenizer (Bertin Technologies, Montigny-le-Bretonneux, France). Then, proteins were digested following the filter-aided FASP protocol described by Wisniewski [84] with minor modifications. Trypsin was added in a trypsin:protein ratio of 1:50, and the mixture was incubated overnight at 37 °C, dried out in a RVC2 25 speedvac concentrator (Martin Christ Gefriertrocknungsanlagen GmbH, Osterode am Harz, Germany), and resuspended in 0.1% formic acid (FA). Peptides were desalted and resuspended in 0.1% FA using C18 stage tips (Merck Millipore, Burlington, MA, USA). Later, samples were analyzed in a novel hybrid trapped ion mobility spectrometry, quadruple time of flight mass spectrometer (timsTOF Pro with PASEF, Bruker, Billerica, MA, USA), coupled online to a nanoElute liquid chromatography (Bruker, Billerica, MA, USA). This mass spectrometer takes advantage of a novel scan mode termed parallel accumulation–serial fragmentation (PASEF), which multiplies the sequencing speed without any loss in sensitivity [85], and has been proven to provide outstanding analytical speed and sensibility for proteomics analyses [86]. The sample (200 ng) was directly loaded in a 15 cm Bruker nanoelute FIFTEEN C18 analytical column (Bruker, Billerica, MA, USA), and resolved at 400 nl/min with a 100 min gradient. The column was heated to 50 °C using an oven. These experiments were performed at the Centre for Cooperative Research in Biosciences (CIC bioGUNE).

#### 4.9.2. Bioinformatics Analysis

The raw files obtained by the MS/MS instrument were analyzed using MaxQuant (https://www.nature.com/articles/s41592-018-0018-y (accessed on 30 November 2021)) in a Linux cluster.

The statistical analysis includes comparisons two to two, among the three conditions. Specifically: MCAO vs. sham, MCAO-Ngb vs. MCAO, and MCAO-Ngb vs. sham. All these comparisons showed the differential protein expression induced either by stroke, by Ngb treatment, or by both.

For ordinary differential analysis and missing data imputation, we used the Bioconductor package DEP (https://www.biorxiv.org/content/10.1101/668863v1.full (accessed on 30 November 2021)). This package provides an integrated analysis workflow for robust and reproducible analysis of mass spectrometry proteomics data for differential protein expression or differential enrichment. Thus, the quantitation matrix performed using DEP allowed us to obtain the different trends for proteins along the three different experimental groups. Briefly, the steps followed using DEP software involved a series of filters to detect only the most variable proteins. In particular, we used the filter_missval DEP function: with the option “thr = 0”, where only proteins that were identified in all replicates of at least one condition were selected. After data normalization (variance stabilizing transformation), missing data imputation was performed using random draws from a Gaussian distribution centered around a q-value = 0.01. Finally, after obtaining the differential expression analysis for the three comparisons under study, a *p*-value under 0.02 and Log2 fold change above 0.5 were used as threshold.

Then, the data were analyzed using different statistical approaches. First, an unsupervised classification algorithm, hierarchical clustering and k-means clustering, was used, looking for groups of proteins acting in a coordinated way. Second, an analysis of gene ontology (GO) and pathways enrichment using STRING (https://www.ncbi.nlm.nih.gov/pmc/articles/PMC6323986/ (accessed on 30 November 2021)) was performed. And third, a pathway projection of the proteins of interest was performed (KEGG, Reactome).

### 4.10. Statistical Analysis

The statistical analysis for the proteomics studies has been performed, as just mentioned, using the MaxQuant and DEP package. To analyze the other parameters studied (i.e., neurological outcomes, infarct volumes, TBARS, and NOx), the SPSS package (SPSS Inc., Chicago, IL, USA) was used. Data are expressed as means ± SD. Statistical comparisons were made using the Levene test to determine the homoscedasticity, and the Student’s t-test to compare the means among the two experimental groups. Statistical significance was set at *p* < 0.05.

## 5. Conclusions

Ngb-NPs systemically injected in a rat model of stroke (MCAO) can cross the BBB, and are endocytosed by neurons of the infarcted area, triggering a neuroprotective action after 24 h of reperfusion.

MCAO-Ngb animals showed higher survival rates, as well as better behavioral scores and improved histological status, although no changes either in the infarct volume or in the oxidative and nitrosative stress assessments were found with the treatment.

The proteomic changes induced by stroke, and the treatment with Ngb-NPs indicated changes in some biological processes, such as endocytosis, cytoskeletal remodeling, or in some metabolic routes; all of them related to damage and restorative mechanisms involved in dendritogenesis, neuritogenesis, or sinaptogenesis.

## Figures and Tables

**Table 1:** Enrichments for the groups of proteins as they appear in the hierarchical cluster at Figure 12.

Cluster	Category	Term	NumGenes	Background	Genes	*p*-Value	FDR	Description
1	Function	GO.0004721	3	83	*Ppm1f, Ppm1h, Lhpp*	1.80 × 10^−4^	0.0113	phosphoprotein phosphatase activity
1	Function	GO.0004812	2	21	*Yars, Gars*	3.90 × 10^−4^	0.0130	aminoacyl-tRNA ligase activity
1	Function	GO.0005546	2	35	*Apba1, Apba2*	1.00 × 10^−4^	0.0149	phosphatidylinositol-4,5-bisphosphate binding
1	Function	GO.0004722	2	30	*Ppm1f, Ppm1h*	7.60 × 10^−4^	0.0149	protein serine/threonine phosphatase activity
1	Function	GO.0001540	2	28	*Apba1, Apba2*	6.70 × 10^−4^	0.0149	amyloid-beta binding
1	Function	GO.0003779	3	189	*Ermn, Coro1b, Wasf1*	1.80 × 10^−4^	0.0153	actin binding
1	Function	GO.0033218	3	228	*Apba1, Apba2, Mag*	3.10 × 10^−4^	0.0218	amide binding
1	Function	GO.0051015	2	76	*Ermn, Coro1b*	4.40 × 10^−4^	0.0269	actin filament binding
2	Function	GO.0003779	4	189	*Wasl, Tmod1, Map1a, Shroom2*	2.40 × 10^−4^	0.0075	actin binding
2	KEGG	rno04144	4	252	*Sh3kbp1, Wasl, Sh3glb1, Sh3glb2*	6.90 × 10^−4^	0.0255	Endocytosis
2	KEGG	rno04141	3	157	*Prkcsh, Dnajc5, Stub1*	2.00 × 10^−4^	0.0378	Protein processing in endoplasmic reticulum
3	Function	GO.0051020	5	181	*Pfn1, Pick1, Madd, Dennd5a, Exoc5*	6.50 × 10^−5^	0.0114	GTPase binding
3	Function	GO.0017112	2	8	*Madd, Dennd5a*	2.30 × 10^−4^	0.0198	Rab guanyl-nucleotide exchange factor activity
3	Function	GO.0017016	4	136	*Pfn1, Madd, Dennd5a, Exoc5*	2.90 × 10^−4^	0.0198	Ras GTPase binding
3	Function	GO.0016863	2	9	*Mif, Eci2*	2.80 × 10^−4^	0.0198	intramolecular oxidoreductase activity, transposing C=C bonds
3	Function	GO.0035255	2	31	*Pick1, Rab4a*	2.60 × 10^−3^	0.0482	ionotropic glutamate receptor binding
4	Function	GO.0052745	2	12	*Ocrl, Synj1*	2.20 × 10^−4^	0.0044	inositol phosphate phosphatase activity
4	Function	GO.0017016	4	136	*Rab3gap2, Ocrl, Kalrn, Stxbp5*	6.89 × 10^−5^	0.0044	Ras GTPase binding
4	Function	GO.0005096	3	90	*Rab3gap2, Ocrl, Stxbp5*	4.30 × 10^−4^	0.0044	GTPase activator activity
4	Function	GO.0004439	2	5	*Ocrl, Synj1*	5.07 × 10^−5^	0.0044	phosphatidylinositol-4,5-bisphosphate 5-phosphatase activity
4	Function	GO.0005088	2	35	*Rab3gap2, Kalrn*	1.60 × 10^−3^	0.0103	Ras guanyl-nucleotide exchange factor activity
4	Function	GO.0017048	2	55	*Ocrl, Kalrn*	3.70 × 10^−3^	0.0210	Rho GTPase binding
4	Function	GO.0017137	2	57	*Rab3gap2, Stxbp5*	3.90 × 10^−3^	0.0211	Rab GTPase binding

**Table 2:** Enrichment for all proteins which underwent significant changes (*p* < 0.05).

Category	Term	NumGenes	Background	Genes
Function	GO.0051020	19	181	*Dnm1l, Rab3gap2, Ocrl, Pfn1, Arfgef1, Rapgef4, Vcl, Pick1, Madd, Dennd5a, Bin1, Ngef, Exoc8, Myo1c, Anxa2, Kalrn, Stxbp5, Exoc5, Wasf1*
Function	GO.0017016	16	136	*Dnm1l, Rab3gap2, Ocrl, Pfn1, Rapgef4, Vcl, Madd, Dennd5a, Ngef, Exoc8, Myo1c, Anxa2, Kalrn, Stxbp5, Exoc5, Wasf1*
Function	GO.0003779	18	189	*Marcks, Pfn1, Hpca, Wasl, Tmod1, Tmod2, Vcl, Cnn3, Pick1, Cap1, Map1a, Tpm5, Myo1c, Ermn, Coro1b, Anxa2, Shroom2, Wasf1*
Function	GO.0050662	16	192	*Abat, Dld, Cryl1, Hmgcl, Txnrd1, Ivd, Idh3a, Ldhb, Etfa, Tkt, Eci2, Got1, Th, Acox1, Phgdh, Fasn*
Function	GO.0050839	11	96	*Calr, Grin2b, Nptn, Src, Peta3, Gfap, Cadm2, Lphn1, P4hb, Nlgn2, Cd151*
Function	GO.0032550	16	203	*Ran, Dnm1l, Arf2, Fkbp4, Hsp90aa1, Arf5, Ehd2, Rap2b, Gnal, Gnai1, Arl2, Prps1, LOC314140, Rab4a, Ehd1, Ak4*
Function	GO.0032561	16	212	*Ran, Dnm1l, Arf2, Fkbp4, Hsp90aa1, Arf5, Ehd2, Rap2b, Gnal, Gnai1, Arl2, Prps1, LOC314140, Rab4a, Ehd1, Ak4*
Function	GO.0015631	14	170	*Dnm1l, Dpysl2, Cnn3, Map1a, Stmn1, Tppp3, Kif5b, Prune, Myo1c, Map4, Chp1, Gphn, Mapre2, Eml1*
Function	GO.0033218	16	228	*Calr, Cltc, Dld, Rnpep, Gria3, Hmgcl, Ctsb, Tpp2, Apba1, Eci2, Apba2, Itm2b, Mag, Apoe, Hspd1, Fasn*
Function	GO.0051015	9	76	*Tmod1, Vcl, Pick1, Tpm5, Myo1c, Ermn, Coro1b, Anxa2, Shroom2*
Function	GO.0000287	10	99	*Ran, Enoph1, Hmgcl, Prpsap1, Idh3a, Tkt, Lhpp, Ephx2, Prps1, LOC314140*
Function	GO.0009055	7	46	*Dld, Sdhb, Txnrd1, Cyb5b, Cyb5a, Etfa, Etfb*
Function	GO.0005525	14	199	*Ran, Dnm1l, Arf2, Fkbp4, Hsp90aa1, Arf5, Ehd2, Rap2b, Gnal, Gnai1, Arl2, Rab4a, Ehd1, Ak4*
Function	GO.0031072	8	66	*Tfam, Cltc, Fkbp4, Sgtb, St13, Hnrpk, Hspa9, Bax*
Function	GO.0005088	6	35	*Rab3gap2, Rapgef4, Madd, Dennd5a, Ngef, Kalrn*
Function	GO.0051082	6	37	*Calr, Hsp90aa1, St13, Hspa9, Hsp90b1, Hspd1*
Function	GO.0016874	8	74	*Farsa, Yars, Gars, Uba5, Acsl3, Sae1, Pars2, Ctps2*
Function	GO.0005085	7	57	*Rab3gap2, Arfgef1, Rapgef4, Madd, Dennd5a, Ngef, Kalrn*
Function	GO.0008017	10	126	*Dnm1l, Dpysl2, Cnn3, Map1a, Kif5b, Myo1c, Map4, Chp1, Mapre2, Eml1*
Function	GO.0004749	3	5	*Prpsap1, Prps1, LOC314140*
Function	GO.0019003	6	44	*Ran, Rap2b, Gnai1, Prps1, LOC314140, Rab4a*
Function	GO.0001540	5	28	*Gria3, Apba1, Apba2, Itm2b, Apoe*
Function	GO.0016616	8	84	*Cryl1, Akr1b1, Idh3a, Ldhb, Fam213b, Hsd17b4, Phgdh, Fasn*
Function	GO.1902936	6	46	*Wipi2, Pfn1, Cadps, Apba1, Apba2, Anxa2*
Function	GO.0017160	3	6	*Exoc8, Myo1c, Exoc5*
Function	GO.0005080	5	30	*Src, Pick1, C1qbp, Akt3, Srsf2*
Function	GO.1901981	7	68	*Wipi2, Gap43, Pfn1, Cadps, Apba1, Apba2, Anxa2*
Function	GO.0042578	12	191	*Ppm1f, Enoph1, Ppm1e, Ocrl, Ppm1h, Pde4b, Ptpre, Lhpp, Ephx2, Prune, Synj1, Plcl1*
Function	GO.0016667	5	32	*Dld, Txnrd1, Pcyox1, Txnl1, P4hb*
Function	GO.0016791	10	139	*Ppm1f, Enoph1, Ppm1e, Ocrl, Ppm1h, Ptpre, Lhpp, Ephx2, Prune, Synj1*
Function	GO.0043021	6	53	*Abcf1, Eif2s1, MGC94335, Hnrpk, C1qbp, ENSRNOG00000037607*
Function	GO.0005546	5	35	*Pfn1, Cadps, Apba1, Apba2, Anxa2*
Function	GO.0017112	3	8	*Rab3gap2, Madd, Dennd5a*
Function	GO.0030165	7	75	*Lin7a, Gria3, Dlgap3, Apba1, Grm5, Mpp2, Kidins220*
Function	GO.0035091	8	98	*Wipi2, Gap43, Pfn1, Cadps, Apba1, Apba2, Anxa2, Ap2a2*
Function	GO.0031625	9	123	*Dnm1l, Calr, Sh3kbp1, Src, Ckb, Bag5, Tsg101, Vcp, Nploc4*
Function	GO.0030246	10	148	*Nptx2, Calr, Tkt, Pgls, Mag, Prps1, Man2c1, Lphn1, LOC314140, Vcan*
Function	GO.0019888	5	36	*Ppp2r2c, Nsfl1c, rCG_47223, Ppp1r1b, Ppp2r5b*
Function	GO.0017048	6	55	*Ocrl, Pfn1, Vcl, Ngef, Kalrn, Wasf1*
Function	GO.0016887	11	177	*Abcf1, Hsp90aa1, Psmc2, Pcyox1, Anxa1, Kif5b, Myo1c, Vcp, Snrnp200, Dync1h1, Lonp1*
Function	GO.0004812	4	21	*Farsa, Yars, Gars, Pars2*
Function	GO.0016668	3	9	*Dld, Txnrd1, Txnl1*
Function	GO.0015643	3	9	*Nefh, Lphn1, Nfm*
Function	GO.0017137	6	57	*Dnm1l, Rab3gap2, Madd, Dennd5a, Anxa2, Stxbp5*
Function	GO.0005543	11	180	*Marcks, Wipi2, Gap43, Pfn1, Cadps, Apba1, Apba2, Anxa1, Anxa2, Apoe, Ap2a2*
Function	GO.0019905	6	58	*Cplx2, Stx6, Scfd1, Rab4a, Stxbp5, Cplx1*
Function	GO.0048306	5	39	*Cplx2, Tsg101, Anxa1, Anxa2, Chp1*
Function	GO.0042277	11	187	*Calr, Cltc, Rnpep, Gria3, Ctsb, Tpp2, Apba1, Apba2, Itm2b, Apoe, Hspd1*
Function	GO.0051087	5	41	*Bag5, Sgtb, St13, Hspa9, Bax*
Function	GO.0043531	4	24	*Prps1, Vcp, LOC314140, Lonp1*
Function	GO.0000149	7	84	*Cplx2, Exoc3, Stx6, Scfd1, Rab4a, Stxbp5, Cplx1*
Function	GO.0004427	2	2	*Lhpp, Prune*
Function	GO.0001018	2	2	*Tfam, Lonp1*
Function	GO.0043022	4	25	*Abcf1, Eif2s1, MGC94335, C1qbp*
Function	GO.0051117	5	43	*Nsfl1c, Hnrpk, Rab4a, Nploc4, Ufd1l*
Function	GO.0008022	8	115	*Cltc, Src, Pick1, Sgtb, Sae1, Myo1c, Synj1, Hpcal4*
Function	GO.0008233	14	299	*Dpp10, Ncstn, Cpq, Rnpep, Nrd1, Scrn1, Ctsb, Sec11a, Tpp2, Psmb2, Pmpca, MGC109340, Arxes2, Lonp1*
Function	GO.0016651	5	49	*Dld, Txnrd1, Nqo1, Txnl1, ND4*
Function	GO.0005178	5	49	*Calr, Peta3, Gfap, P4hb, Cd151*
Function	GO.0051287	5	50	*Dld, Cryl1, Idh3a, Ldhb, Phgdh*
Function	GO.0016903	4	32	*Dld, Akr1b1, Aldh1b1, Bckdha*
Function	GO.0032564	2	4	*Hsp90aa1, St13*
Function	GO.0030984	2	4	*Ctsb, C1qbp*
Function	GO.0019834	2	4	*Anxa1, Anxa2*
Function	GO.0050660	5	54	*Dld, Txnrd1, Ivd, Etfa, Acox1*
Function	GO.0016829	7	105	*Cd38, Hmgcl, Echdc1, Hsd17b4, Got1, Adcy5, Fasn*
Function	GO.0051400	2	5	*Dnm1l, Bax*
Function	GO.0036435	2	5	*Vcp, Ufd1l*
Function	GO.0017075	3	18	*Cplx2, Stxbp5, Cplx1*
Function	GO.0016627	4	36	*Sdhb, Ivd, Acox1, Fasn*
Function	GO.0005030	2	5	*Ntrk2, Sort1*
Function	GO.0004439	2	5	*Ocrl, Synj1*
Function	GO.0003924	8	135	*Ran, Dnm1l, Rap2b, Gnal, Gnai1, Gng7, Arl2, Rab4a*
Function	GO.0045182	5	58	*Abcf1, Eif2s1, Cirbp, Eef1d, Eif4e*
Function	GO.0031406	8	139	*Grin2b, Hmgcl, Got1, Th, Mag, C1qbp, Vcan, Acox1*
KEGG	rno04144	28	252	*Vps37b, Cltc, Sh3kbp1, Snx6, Arfgef1, Wasl, Arf5, Ehd4, Arpc4, Rab11fip2, Src, Ehd2, Bin1, Sh3glb1, Tsg101, Ap2a1, Ist1, Mvb12b,*
				*Sh3glb2, Kif5b, Vps35, Rab11fip5, Rab4a, Ehd1, Hgs, Ap2a2, Vps26b, Smap1*
KEGG	rno04141	20	157	*Calr, Skp1, Hsp90aa1, Gcs1, Nsfl1c, Fbxo2, Eif2s1, Sec13, Prkcsh, Dnajc5, Stub1, Bax, Wfs1, Hsp90b1, Sec24b, Vcp, P4hb, Nploc4, Ssr4, Ufd1l*
KEGG	rno04728	15	122	*Ppp2r5a, Ppp2r5e, Ppp2r2c, Gria3, Grin2b, Kif5b, Gnal, Gnai1, Gng7, Th, Ppp1r1b, Adcy5, Akt3, Gng4, Ppp2r5b*
KEGG	rno01200	13	112	*Sdhc, Dld, Acss1, Sdhb, Idh3a, Tkt, Got1, Pgls, Prps1, Cs, LOC314140, Phgdh, Acss2*
KEGG	rno04727	11	86	*Abat, Gabrg2, Adcy1, Src, Gnai1, Gng7, Adcy5, Plcl1, Slc12a5, Gphn, Gng4*
KEGG	rno00640	7	30	*Abat, Dld, Acss1, Echdc1, Ldhb, Bckdha, Acss2*
KEGG	rno00970	8	45	*Farsa, Yars, Gars, Nars, Hars, Mars, Iars2, Pars2*
KEGG	rno05016	13	181	*Tfam, Sdhc, Cltc, Ndufa12, Sdhb, Grin2b, Ndufb9, Tgm2, Ap2a1, Grm5, Bax, LOC685596, Ap2a2*
KEGG	rno04723	11	144	*Gabrg2, Ndufa12, Gria3, Adcy1, Ndufb9, Grm5, Gnai1, Gng7, ND4, Adcy5, Gng4*
KEGG	rno05132	8	79	*Pfn1, Wasl, Arpc4, Dync1li2, Actb, Klc2, Wasf1, Dync1h1*
KEGG	rno05030	6	46	*Grin2b, Gnai1, Th, Ppp1r1b, Adcy5, Gpsm1*
KEGG	rno05012	10	134	*Sdhc, Ndufa12, Sdhb, Ndufb9, Gnal, Gnai1, Th, ND4, Adcy5, LOC685596*
KEGG	rno03050	6	46	*Psmd6, Psmb2, Psmc2, Psmd1, Psmd4, Psmd3*
KEGG	rno00190	10	130	*Ppa1, Atp6v1g2, Sdhc, Ndufa12, Sdhb, Ndufb9, Ppa2, Lhpp, ND4, LOC685596*
KEGG	rno00020	5	29	*Sdhc, Dld, Sdhb, Idh3a, Cs*
KEGG	rno00280	6	51	*Abat, Dld, Hmgcl, Ivd, Aldh1b1, Bckdha*
KEGG	rno05100	7	73	*Cltc, Wasl, Arpc4, Src, Vcl, Actb, Wasf1*
KEGG	rno01230	7	73	*Idh3a, Tkt, Got1, Prps1, Cs, LOC314140, Phgdh*
KEGG	rno00620	5	35	*Dld, Acss1, Aldh1b1, Ldhb, Acss2*
KEGG	rno04721	6	60	*Cplx2, Atp6v1g2, Cltc, Ap2a1, Cplx1, Ap2a2*
KEGG	rno04261	9	133	*Ppp2r5a, Ppp2r5e, Ppp2r2c, Rapgef4, Adcy1, Gnai1, Adcy5, Akt3, Ppp2r5b*
KEGG	rno04146	7	82	*Hmgcl, Scp2, Acsl3, Hsd17b4, Eci2, Ephx2, Acox1*
KEGG	rno04120	9	130	*Skp1, Fbxo2, Ube2o, Sae1, Ube4a, Cul4a, Stub1, Ddb1, Cul4b*
KEGG	rno00010	6	59	*Dld, Acss1, Bpgm, Aldh1b1, Ldhb, Acss2*
KEGG	rno04724	8	109	*Gria3, Adcy1, Grin2b, Grm5, Gnai1, Gng7, Adcy5, Gng4*
KEGG	rno05010	10	164	*Sdhc, Ncstn, Ndufa12, Sdhb, Grin2b, Ndufb9, Lrp1, App, LOC685596, Apoe*
KEGG	rno04714	12	221	*Sdhc, Ndufa12, Sdhb, Adcy1, Ndufb9, Acsl3, Slc25a20, ND4, Smarcd1, Actb, Adcy5, LOC685596*
KEGG	rno05032	7	88	*Gabrg2, Pde4b, Adcy1, Gnai1, Gng7, Adcy5, Gng4*
KEGG	rno04713	7	90	*Gria3, Adcy1, Grin2b, Gnai1, Gng7, Adcy5, Gng4*
KEGG	rno04071	8	116	*Ppp2r5a, Ppp2r5e, Ppp2r2c, Gnai1, Bax, Akt3, Pdpk1, Ppp2r5b*
KEGG	rno00030	4	29	*Tkt, Pgls, Prps1, LOC314140*
KEGG	rno05169	11	206	*Ran, Cd38, Psmd6, Psmc2, Psmd1, Vim, Psmd4, Psmd3, Snw1, Akt3, ENSRNOG00000025731*
KEGG	rno04926	8	122	*Adcy1, Src, Gnai1, Gng7, Adcy5, Col4a1, Akt3, Gng4*
KEGG	rno04915	8	122	*Fkbp4, Hsp90aa1, Adcy1, Src, Gnai1, Hsp90b1, Adcy5, Akt3*
KEGG	rno05034	8	130	*Grin2b, Gnai1, Gng7, Th, Ppp1r1b, Ntrk2, Adcy5, Gng4*
KEGG	rno04024	10	188	*Pde4b, Gria3, Rapgef4, Adcy1, Grin2b, Gnai1, Ppp1r1b, Adcy5, Acox1, Akt3*
KEGG	rno01210	3	17	*Idh3a, Got1, Cs*

**Table 3:** Bederson’s neurological score system. Total score ranges from 0 to 9.

Feature	Score
Spontaneous activity	Moving and exploring = 0 Moving without exploring = 1 No moving or moving only when pulled by the tail = 2
Left drifting during displacement	None = 0 Drifting only when elevated by the tail and pushed or pulled = 1 Spontaneous drifting = 2 Circling without displacement, or spinning = 3
Resistance to left forepaw stretching	Stretching not allowed= 0 Stretching allowed after some attempts = 1, No resistance = 2
Parachute reflex	Symmetrical = 0 Asymmetrical = 1 Contralateral forelimb retracted = 2

## Supplementary Materials

The following are available online at https://www.mdpi.com/article/10.3390/ijms23010247/s1.
